# Descriptive review of tuberculosis surveillance systems across the circumpolar regions

**DOI:** 10.3402/ijch.v75.30322

**Published:** 2016-04-26

**Authors:** Annie-Claude Bourgeois, Tammy Zulz, Bolette Soborg, Anders Koch, on behalf of the International Circumpolar Surveillance – Tuberculosis Working Group

**Affiliations:** 1 Centre for Communicable Diseases and Infection Control, Public Health Agency of Canada, Ottawa, ON, Canada; 2 Arctic Investigations Program, Division of Preparedness and Emerging Infections, National Center for Emerging and Zoonotic Diseases, Centers for Disease Control and Prevention, Anchorage, AK, USA; 3 Department of Epidemiology Research, Statens Serum Institut, Copenhagen, Denmark

**Keywords:** surveillance, tuberculosis, circumpolar, International Surveillance Circumpolar – Tuberculosis Working Group

## Abstract

**Background:**

Tuberculosis is highly prevalent in many Arctic areas. Members of the International Circumpolar Surveillance Tuberculosis (ICS-TB) Working Group collaborate to increase knowledge about tuberculosis in Arctic regions.

**Objective:**

To establish baseline knowledge of tuberculosis surveillance systems used by ICS-TB member jurisdictions.

**Design:**

Three questionnaires were developed to reflect the different surveillance levels (local, regional and national); all 3 were forwarded to the official representative of each of the 15 ICS-TB member jurisdictions in 2013. Respondents self-identified the level of surveillance conducted in their region and completed the applicable questionnaire. Information collected included surveillance system objectives, case definitions, data collection methodology, storage and dissemination.

**Results:**

Thirteen ICS-TB jurisdictions [Canada (Labrador, Northwest Territories, Nunavik, Nunavut, Yukon), Finland, Greenland, Norway, Sweden, Russian Federation (Arkhangelsk, Khanty-Mansiysk Autonomous Okrug, Yakutia (Sakha Republic), United States (Alaska)] voluntarily completed the survey – representing 2 local, 7 regional and 4 national levels. Tuberculosis reporting is mandatory in all jurisdictions, and case definitions are comparable across regions. The common objectives across systems are to detect outbreaks, and inform the evaluation/planning of public health programmes and policies. All jurisdictions collect data on confirmed active tuberculosis cases and treatment outcomes; 11 collect contact tracing results. Faxing of standardized case reporting forms is the most common reporting method. Similar core data elements are collected; 8 regions report genotyping results. Data are stored using customized programmes (n=7) and commercial software (n=6). Nine jurisdictions provide monthly, bi-annual or annual reports to principally government and/or scientific/medical audiences.

**Conclusion:**

This review successfully establishes baseline knowledge on similarities and differences among circumpolar tuberculosis surveillance systems. The similarity in case definitions will allow for description of the epidemiology of TB based on surveillance data in circumpolar regions, further study of tuberculosis trends across regions, and recommendation of best practices to improve surveillance activities.

Surveillance is a key component of efforts to control and eradicate tuberculosis (TB) globally (1,2). While a standardized approach to TB surveillance does not exist, the World Health Organization (WHO) has put forth a TB surveillance checklist of standards and benchmarks in an attempt to help countries and regions identify gaps in their current surveillance systems and bolster their ability to attain accurate measures of annual TB cases and deaths (3). Most jurisdictions however, even within a country, have developed their own surveillance programmes based on their unique infrastructure and needs as well as the varied political interests, geography and resources for each region (4). Variations across surveillance systems create the potential for inconsistencies in data and thus present challenges when comparing data and trends across regions/globally.

In 1999, the International Circumpolar Surveillance (ICS) system was established by the Arctic Council's Sustainable Development Working Group to create an infectious disease surveillance network throughout Arctic jurisdictions (5). In 2006, ICS representatives from Canada, Greenland and Alaska met to establish a TB surveillance subgroup of the ICS network, as it was recognized that TB continues to be a significant health problem in the circumpolar region (6).

The circumpolar region comprises Greenland (that has self-government but is part of the Danish Kingdom), Iceland (as a sovereign country) and northern political-administrative regions of countries including the United States of America (USA), Canada, Norway, Sweden, Finland, and the Russian Federation (7). For these areas, health services are organized on either local, regional or national levels. For the purpose of this review, all of these areas are referred to as “jurisdiction” and are classified according to their self-identified health system organization (e.g. local jurisdiction).

To date, the ICS-TB Working Group includes jurisdictional representatives from northern Canada, Finland, Greenland, Norway, Sweden, Russian Federation and the USA (Fig. 1). The mandate of ICS-TB is to increase international knowledge on TB epidemiology-related issues in the circumpolar region as well as to support further epidemiologic projects and provide evidence to inform policy decisions, programme design and evaluation in the jurisdictions of the circumpolar region.

**Fig. 1:**
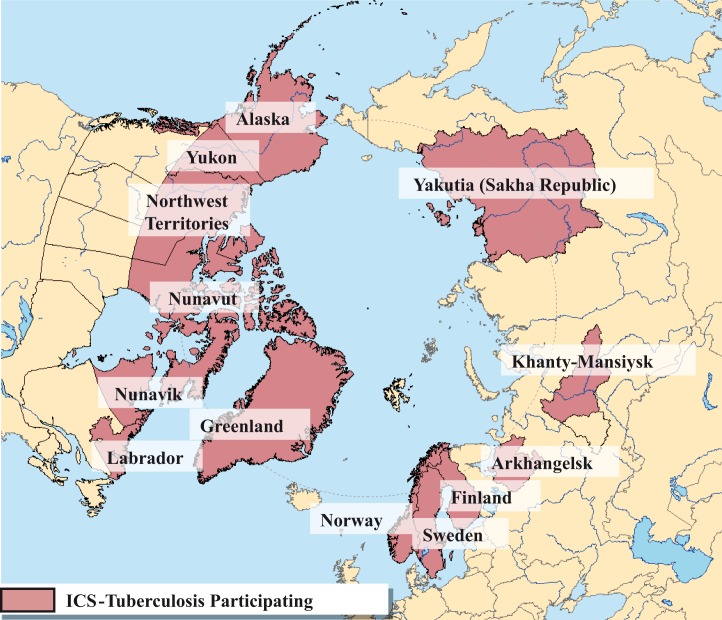
Participating jurisdictions of the International Circumpolar Surveillance Tuberculosis Working Group.

In order to accurately compare TB trends across member jurisdictions and to establish baseline knowledge on data recording and reporting, members of the ICS-TB Working Group reviewed the TB surveillance systems of ICS-TB jurisdictions. The goal of this project was to describe the general characteristics of the different TB surveillance systems used by ICS-TB jurisdictions to better inform and support future data analyses. To the best of our knowledge, this is the first descriptive review that compares TB surveillance systems characteristics that focus solely on circumpolar jurisdictions.

## Methods

Questions were developed based on previously established evaluation and component criteria of public health surveillance systems (4,8,9). Three versions of a questionnaire were created to reflect the surveillance levels, local, regional and national within the member jurisdictions. We defined the surveillance levels as follows: a) A local jurisdiction collects primary data on patients with diagnosed or suspected TB for a local health unit, TB clinics and/or health care providers and the data are sent to a secondary level of surveillance for collation, analysis and so on. b) A regional jurisdiction receives data from local jurisdictions where collation and analysis may be performed with reporting back to the local level. Data are sent to the national level. c) A national level jurisdiction receives data from local and/or regional jurisdictions; collation and analysis are performed and reporting may be made back to the local and or regional level. Data may be aggregated with other jurisdictions for national reporting and reporting internationally, if applicable. The number of questions varied from 31 (national) to 36 (local) with 24 questions being identical in all 3 questionnaires. The remaining questions were specific to each jurisdictional level.

Questions were closed-ended, where possible, for better comparability of the answers. Specific aspects investigated included: a) activities and objectives, b) reporting activities, c) case identification methods, d) data collection, e) data storage and f) data dissemination. The questionnaire outlined 6 main activities and objectives of TB surveillance systems as proposed by the CDC on the uses of surveillance data (8).

All participants also had the option to provide a flow chart of their organizational structure or additional documents on their respective surveillance systems to complement the information provided in the questionnaire.

All 3 questionnaires were forwarded by electronic mail to the official representative of each of the 15 ICS-TB member jurisdictions. Participation in this project was voluntary. The respondent was requested to identify the jurisdictional level that pertained to the surveillance system in their region and complete the appropriate questionnaire.

Preliminary review of the completed questionnaires revealed that some questions were interpreted differently by the participants and that additional information was needed for meaningful analysis and interpretation of the questionnaire responses. The questionnaires were revised and sent back to participants for their completion.

## Results

Thirteen of the fifteen ICS-TB jurisdictions completed the initial version of the questionnaire; of these 13, 12 returned the revised version and 1 provided the additional information by email. Two jurisdictions completed the questionnaires for “local” [Labrador and Yukon (Canada)], 7 for “regional” [Nunavik, Nunavut and Northwest Territory (Canada); Arkhangelsk, Khanty-Mansiysk Autonomous Okrug and Yakutia (Sakha Republic) – (Russian Federation); and Alaska (USA)] and 4 for “national” (Finland, Greenland, Norway and Sweden). Nine jurisdictions provided flow charts and/or additional comments or documents (Alaska, Arkhangelsk, Finland, Greenland, Khanty-Mansiysk Autonomous Okrug, Northwest Territories, Nunavik, Sweden and Yukon).

Definitions of active TB cases were comparable across the 13 jurisdictions; 5 use the WHO standards (10) and 8 use slightly modified WHO standards. Briefly, a case of active TB is deemed laboratory confirmed if *Mycobacterium tuberculosis* complex (*M. tuberculosis*, *M. bovis*, *M. africanum*, *M. canetti* and *M. microti*) is demonstrated on culture or by newer methods such as molecular line probe assay or a sputum specimen positive for acid-fast bacilli. In participating jurisdictions, a clinically diagnosed or probable case refers to a case for which, in absence of bacteriological proof, there were either chest radiographic changes compatible with active TB and/or treatment has been initiated. All jurisdictions identify active cases through laboratory confirmation and/or clinical diagnosis by chest X-ray (Nunavik and Alaska also require compatible clinical signs with a positive X-ray). National reporting systems allow for the determination of whether a case is new or relapsed as a result of previous treatment failure, consistent with the WHO definitions for 3 regions. This information was not available for 1 jurisdiction.

All 13 jurisdictions reported outbreak identification and control, informing programme planning and policy development, and contributing to the evaluation of public health programmes and policies as the main objectives and activities of their TB surveillance systems. Most, but not all, reported that the additional (secondary) objectives of the surveillance systems were: scientific and research purposes (except Labrador, Yukon and Khanty-Mansiysk); generating and maintaining public awareness (except Khanty-Mansiysk); and conducting evaluations to identify gaps and areas of improvement (except Khanty-Mansiysk). For the 4 national systems, TB surveillance systems were designed to meet all 6 objectives and activities listed in the questionnaire.

All TB surveillance systems in the participating jurisdictions are centralized and publically funded. Reporting of laboratory-confirmed active TB cases and treatment outcome is mandatory in all jurisdictions (Table I). Clinically diagnosed cases are reported in 11 jurisdictions (85%). Contact screening results were reportable in all jurisdictions except for Sweden and Finland. Latent TB infection (LTBI) is reportable in 6 (46%) local or regional jurisdictions; in 2 jurisdictions, LTBI is reportable in the context of contact screening only; in 4 jurisdictions, LTBI is reportable regardless of the reason for screening. However, treatment outcomes of LTBI are reportable in 8 (62%) of the jurisdictions, including 1 national (Greenland); of note, LTBI is reportable in some jurisdictions only when prophylactic treatment is initiated.

**Table I:** Surveillance system characteristics and tuberculosis reporting elements for each jurisdiction

			Reportable to TB programme							
			
Jurisdictions		Year TB surveillance system introduced	Clinical active cases	Confirmed active cases	Suspected cases	Treatment outcomes of active cases	Latent TB infections^a^	All latent TB infections^b^	Treatment outcomes for latent TB infections	All contacts screening results^c^
Local	Labrador	–	X	X	X	X	X	X	X	X
	Yukon	2004	X	X	X	X	–	X	X	X
Regional	Khanty-Mansiysk Autonomous Okrug	1998	–	X	–	X	–	–	X	X
	Arkhangelsk	1998	–	X	–	X	X	X	X	X
	Yakutia	2005	X	X	–	X	–	–	X	X
	Nunavik	1990	X	X	–	X	X	–	–	X
	Northwest Territories	–	X	X	X	X	X	X	X	X
	Nunavut	1999	X	X	X	X	X	–	X	X
	Alaska	1950's	X	X	X	X	–	–	–	X
National	Greenland	1956	X	X	–	X	–	–	X	X
	Sweden	1940	X	X	–	X	–	–	–	–
	Finland	1995	X	X	–	X	–	–	–	–
	Norway	1962	X	X	X	X	–	–	–	X

a Individuals with a positive Tuberculin Skin Test (TST) and/or IGRA result – In the context of contact screening only.

b Regardless of the reason for screening.

c From contact tracing, including positive, negative results as well as unknown/not screened.

The estimated proportion of active TB cases reported ranged from 75 to 100% (Table II). All jurisdictions collect case-level data using standardized reporting forms with supporting guidelines and/or data dictionary; 2 regional and 1 national jurisdiction also collect aggregated data (Khanty-Mansiysk, Nunavik and Norway). Data are received using one or multiple data submission methods including electronic submissions that feed into the TB surveillance database (n=5; 42%), faxing (n=8; 62%), emails (n=4; 33%), postal mail (n=7; 54%) or email consultations from clinicians on cases and/or contacts (n=1; 8%). Of all jurisdictions, only 3 of them, Arkhangelsk, Greenland and Sweden, rely solely on electronic submissions (Table II). All local and regional jurisdictions, except Alaska, are provided with a standardized form for data reporting to higher jurisdictional levels. Local jurisdictions report either weekly or on an ad hoc basis to their regional offices, and regional jurisdictions mainly report to the national level on an annual basis.

**Table II:** Data collection and storage for surveillance systems

Jurisdictions		Estimated % of all TB cases captured by system	Case submission methods	Method of storage	Data accessible to others
Local	Labrador	100	Electronic to database, fax	Commercial software	No
	Yukon	100	Fax, mail	Commercial software, in-house/custom software	Available online
Regional	Khanty-Mansiysk Autonomous Okrug	100	Fax, mail	Commercial software, paper-based	Available upon request
	Arkhangelsk	95–98	Electronic to database	In-house/custom software	Partial access
	Yakutia	100	Fax, mail	In-house/custom software	Available upon request
	Nunavik	95–98	Email, fax, other	Commercial software, paper-based	Available upon request
	Northwest Territories	80	Email, fax, mail	In-house/custom software, paper-based	Available upon request
	Nunavut	100	Email, fax, mail	Commercial software, paper-based	No
	Alaska	100	Email, fax, mail	In-house/custom software, paper-based	Available upon request
National	Greenland	75	Electronic to database	Commercial software	No
	Sweden	95–100	Electronic to database	In-house/custom software	Available upon request
	Finland	95	Electronic to database, mail	In-house/custom software	Available upon request, available online, partial access
	Norway	~100	Mail	In-house/custom software	Available upon request, available online

Core data elements reported within the TB surveillance systems are similar across jurisdictions, including demographic information, laboratory results, chest X-ray or computerized tomography (CT) scan (except Finland), PCR test results (except Khanty-Mansiysk) and drug-resistance test results (except Khanty-Mansiysk). Genotyping test results such as mycobacterial interspersed repetitive units (MIRU) or spoligotype are reported in Labrador, Yukon, Nunavik, Nunavut, Alaska and all 4 national jurisdictions.

Usually only positive laboratory results are reported, but for 4 jurisdictions (Arkhangelsk, Khanty-Mansiysk Autonomous Okrug, Yakutia and Finland) both negative and positive laboratory results (smear and culture) are communicated to these jurisdictional TB surveillance programmes. Risk factors, social determinants of health and/or co-morbidity for active cases are collected in 9 jurisdictions (69%), with Norway being the only national jurisdiction collecting such information. Testing for human immunodeficiency virus (HIV) status for all active TB cases is done or requested in all local and regional jurisdictions except Nunavut and 1 national jurisdiction (Norway). When available, results are reported directly to the TB surveillance programme in those jurisdictions (except Nunavik). Although testing and results are not reported directly to the TB programme in Finland, there is cross-matching between the HIV and TB databases.

Social determinants of health, such as data on homelessness, incarceration, smoking status, substance abuse, steroid use, diabetes diagnosis and others, are collected in all local and regional jurisdictions except Nunavut and 1 national jurisdiction (Norway).

Data are stored using commercial software such as Microsoft Excel™ or Access™ (n=6; 46%) and/or in-house/customized programmes (n=7; 54%) (Table II). Four regional jurisdictions (31%) also maintain paper-based records in addition to an electronic system; when writing this article, Alaska was transitioning from a completely paper-based system to a customized electronic system. A unique case identifier number for active TB cases is used in all jurisdictions. In both local jurisdictions (Labrador and Yukon), contact tracing information is collected by paper form. In Yukon, this information is also captured in a computerized database that is not linked to the TB surveillance database. Detection for discrepancies and duplicates in the database is performed for all TB surveillance systems, and these data verification functions are performed automatically (e.g. prompting alert) and/or manually (Greenland only). Data on TB cases, either line-listed and/or aggregated, are available to the public from most jurisdictions, either online (n=3; 23%) and/or upon request (n=7; 54%) (Table II).

Reports using TB surveillance data are produced by all jurisdictions except Yukon (n=11, 92%) mainly annually (n=8; 62%) and/or on an ad hoc basis (n=6; 46%). Of those jurisdictions disseminating data, report audiences are government(s) (n=12/12; 100%), the scientific/medical community (n=9/12; 75%), and/or the general public (n=8/12; 66%).

## Discussion

To our knowledge, this is the first review of the general characteristics, similarities and differences of TB surveillance systems across circumpolar jurisdictions. A similar review of TB surveillance systems was conducted by Mor et al. (4); however, the review focused on low-incidence industrialized countries in Western Europe, the USA, Canada, Australia and New Zealand. Across all surveyed circumpolar jurisdictions, surveillance of TB is a deep-rooted activity of the public health system; reporting of active TB cases and their respective treatment outcome is mandatory in each ICS-TB jurisdiction, aligned with the WHO recommendations, with reporting completeness estimated (by the jurisdictions themselves) to be close to 100%. In addition, and regardless of the surveillance level, all systems are centralized and publically funded.

### System descriptions

Thirteen ICS-TB jurisdictions participated in this project, representing 7 countries in, or with partial geographical area in the circumpolar region. There are notable differences among the jurisdictions participating to ICS-TB in terms of resources, geography and social determinants of health that may affect the efficacy of a TB programme and related surveillance system (11). However, influential factors across jurisdictions outside of TB surveillance systems (e.g. financial resources, remoteness and housing shortages resulting in overcrowding) were not investigated in the survey, and therefore, comments on the impacts of those factors on a TB surveillance system cannot be assessed.

### Case definitions

Although there were slight variations in the definitions provided for active TB cases or in the terminology (e.g. clinically confirmed versus probable cases), identical components were found in all definitions and aligned with the WHO definitions (9). In addition, most of the core data elements collected by the jurisdictions for active TB cases and their resulting treatment outcome are similar and captured in a standardized way via forms. Those similarities allow for the development of harmonized definitions among the ICS-TB jurisdictions for further description of the epidemiology of TB and trends in the circumpolar region.

The purpose of a public health surveillance system varies depending on the public health needs and roles of a defined jurisdiction (8). In all the ICS-TB jurisdictions, the TB surveillance systems’ objectives go beyond and above case management, outbreak identification and disease monitoring, and are linked to programme and policy planning, development and evaluation as reports produced by the jurisdictions target governmental and scientific audiences (12–14). In this era of globalization, and with this goal in mind, there is a potential for the ICS-TB jurisdictions to consider providing recommendations to improve TB surveillance in circumpolar regions.

### Contact tracing and LTBI

A common practice in TB control and prevention is to conduct contact tracing for active cases in order to identify and treat TB-infected contacts (15–17). Despite the challenges faced by most of the circumpolar regions (e.g. limited staff and financial resources), local TB programmes in the ICS-TB jurisdictions and some regional programmes from low-density populations, such as Nunavik, Northwest Territories and others, collect results and information on LTBI and/or contact tracing to ensure appropriate case management and better outbreak prevention. LTBI reporting and monitoring appear to be a lower priority at the national level, as only Greenland collects information (and in this jurisdiction, LTBI is reportable only when isoniazid preventive therapy is initiated). National level interest in collecting LTBI and contact information was not included in this survey; however, given the current WHO recommendations towards TB elimination, there is likely to be increased focus on LTBI monitoring in the future (18).

### Social determinants, risk factors and co-morbidities

The collection of information on social determinants of health, risk factors and co-morbidities for TB (e.g. homelessness, incarceration, smoking status, substance abuse, steroid use, diabetes and HIV) resides mainly in the local and regional circumpolar jurisdictions; however, whether this information was reported to a higher level (i.e. at regional or country level) for those regions was not assessed. None of the ICS-TB national jurisdictions collect those elements, with the exception of Norway where some information on HIV is gathered. In some cases, this information may possibly be captured in different registries linked with the TB surveillance database (4), such as Greenland. The impact of previously identified risk factors for TB and co-morbidities such as HIV or diabetes in the circumpolar region (19,20) may be estimated through this collaboration and may further support programme and policy planning in those jurisdictions.

### Drug resistance

As drug resistant TB has become a global concern and public health priority, monitoring of emerging trends and patterns in anti-tuberculosis drug resistance is a key activity in TB control (3,21). Drug-resistance testing of TB isolates is done across all ICS-TB jurisdictions, and laboratory results are all reported directly from the laboratory to the TB surveillance programme (except Khanty-Mansiysk), therefore reducing the likelihood of errors due to a multilayered reporting system.

### Electronic reporting systems

With computerized technology becoming more available, online reporting systems are being developed and currently 5 ICS-TB jurisdictions, including 3 national ones, have an online reporting system directly linked to the TB surveillance system. Fax remains the most common way of submitting data, but data still need to be entered manually in the electronic database, which increases the likelihood of transcription mistakes and is a time consuming process. However, some jurisdictions have overcome these challenges – Greenland has an electronic reporting system despite the remoteness of most of its communities. The experience and lessons learned on TB surveillance and database development from ICS-TB jurisdictions sharing similar characteristics may be of use to provide recommendations to other ICS-TB regions wishing to move towards electronic submission.

## Limitations

Our study includes some limitations that may affect the interpretation of the results. Each jurisdiction self-identified the appropriate reporting level. Although definitions were provided for the 3 levels, there may be discrepancies in the self-identification process and selection of the appropriate survey. This survey included only ICS-TB member jurisdictions and was not extended to circumpolar jurisdictions outside of the working group, which may have differences in their TB surveillance systems structure and processes. Comparisons were also made from various levels of reporting, regardless of the differences in resources (human, national gross income, etc.), geography and technical and technological capacities; therefore, the interpretation of some results should be made with caution. The questionnaires were not designed to evaluate attributes of the TB surveillance systems or databases (such as data quality, sensitivity or timeliness), and therefore, it is not possible to make system recommendations or to comment on the impacts of incorporating changes in a given surveillance system. This study was cross-sectional, focusing only on the TB surveillance systems and did not take into consideration the progression, improvements or challenges (e.g. resources) of the different surveillance systems and TB programme structures over time.

## Conclusion

Similarities and differences among the circumpolar TB surveillance systems highlighted in this review establish baseline knowledge on data recording and reporting of ICS-TB member jurisdictions and will allow for the contextualization of TB trends across jurisdictions. Although case definitions are similar and consistent with the WHO standards, it will allow for the description of the epidemiology of TB on multilevel surveillance data in circumpolar region and further study of TB trends across regions. Furthermore, the information collected in the survey will serve to guide further discussion within the ICS-TB working group to make recommendations on best practices to improve surveillance activities in circumpolar regions.
